# An inherent instability study using ab initio computational methods and experimental validation of Pb(SCN)_2_ based perovskites for solar cell applications

**DOI:** 10.1038/s41598-020-72210-4

**Published:** 2020-09-17

**Authors:** Jayita Dutta, Mithun Chennamkulam Ajith, Soumya Dutta, Umesh R. Kadhane, Jinesh Kochupurackal B, Beena Rai

**Affiliations:** 1 Physical Sciences Research Area, Tata Research Development and Design Centre, TCS Research, Tata Consultancy Services, 54, B Hadapsar Industrial Estate, Pune, 411013 India; 2 Indian Institute of Technology Madras, Tamil Nadu, Chennai, 600036 India; 3 Indian Institute of Space Science and Technology, Valiamala Road, Valiamala, Trivandrum, Kerala 695547 India

**Keywords:** Materials for devices, Theory and computation, Electronics, photonics and device physics, Materials science, Physics

## Abstract

Perovskite materials with ABX_3_ chemistries are promising candidates for photovoltaic applications, owing to their suitable optoelectronic properties. However, they are highly hydrophilic and unstable in nature, limiting the commercialization of perovskite photovoltaics. Mixed halide ion-doped perovskites are reported to be more stable compared to simple ABX_3_ chemistries. This paper describes ab initio modeling, synthesis, and characterization of thiocyanate doped lead iodide CH_3_NH_3_PbI_(3*−x*)_(SCN)_*x*_ perovskites. Several perovskite chemistries with an increasing concentration of (SCN)^−^ at x = 0, 0.25, 0.49, 1.0, 1.45 were evaluated. Subsequently, ‘n-i-p’ and ‘p-i-n’ perovskite solar device architectures, corresponding to x = 0, 0.25, 0.49, 1.0 thiocyanate doped lead halide perovskite chemistry were fabricated. The study shows that among all the devices fabricated for different compositions of perovskites, p-i-n perovskite solar cell fabricated using CH_3_NH_3_PbI_(3*−x*)_(SCN)_*x*_ perovskite at x = 1.0 exhibited the highest stability and device efficiency was retained until 450 h. Finally, a solar panel was fabricated and its stability was monitored.

## Introduction

Organic–inorganic hybrid halide perovskite materials with ABX_3_ chemistry such as methylammonium lead iodide (CH_3_NH_3_PbI_3_) based perovskite solar cells where methylammonium (CH_3_NH_3_^+^) is at the A site, lead (Pb^2+^) at the B site and halide (X = I^−^, Cl^−^, Br^−^) at the X site, have ruled the photovoltaic industry since 2009^1,2^. The mystery behind the exponential interest in perovskite materials in the field of the photovoltaic industry can be pointed to a continuous unparalleled increase in the efficiency of perovskite-based solar cells from 3.8% to over 20% in less than 5 years duration^3^. Perovskite materials consisting of metal halides have gained interest as a solar material because of their superior optoelectronic properties such as direct bandgap (≈ 1.5 eV), high light absorption coefficient, ambipolar charge transport abilities, long carrier lifetime, and high carrier mobility. Furthermore, they are suitable for active solar materials for different types of photovoltaic architecture^4^. Thus in perovskite solar cells (PSCs), the active perovskite layer not only acts as a light-absorbing material but also acts as an electron and hole transporting material owing to its ambipolar transport abilities. Moreover, synthesis of metal halide perovskites involves cost-effective, easy and simple synthesis methods like one-step or two-step solution processing, or by vapor deposition techniques or by vapor assisted solution processing to ensure uniform film morphology^4^. These advantages make perovskites, superior candidates for photovoltaic applications.

However, despite having excellent optoelectronic properties ideal for photovoltaic applications, organolead halide perovskites are highly hydrophilic in nature and thus are unstable when exposed to ambient moisture. Long term stability of organolead halide perovskites in the ambient environment remains a challenge thus posing the biggest hindrance towards commercialization of perovskite solar cells^4^. Methylammonium lead halide such as CH_3_NH_3_PbI_3_ perovskites readily hydrolyses or ends up forming intermediate hydrate compounds such as (CH_3_NH_3_)_4_PbI_6_:2H_2_O when exposed to a relative humidity of 80%. Perovskite material easily absorbs moisture from the atmosphere and decomposes into precursor salts like (PbX_2_) and CH_3_NH_3_X^5,6^. Due to moisture instability under ambient conditions, CH_3_NH_3_PbX_3_ (MAPbX_3_) films are required to be synthesized in an inert atmosphere inside a glove box to avoid long term exposure of the films to ambient conditions. However, the synthesis of perovskite material inside a glove box under inert conditions does not ensure long term stability when exposed to ambient conditions^4^. Moreover, the intrinsic material properties of organolead halide perovskites make them thermally unstable. The material undergoes significant decomposition when annealed at 85^o^ C under inert conditions. Furthermore, perovskite materials kept exposed to solar radiation for a longer duration, comes in contact with UV rays and undergoes photochemical degradation thereby resulting in distortion of the perovskite structure^7^. Degradation in PSC even occurs due to the elementary migration of ions in the perovskite material^8^. Thus these perovskite materials undergo structural deformation or alteration under thermal stress^9^.

Various approaches have been reported to overcome the stability issues of perovskite material such as the use of water-resistive coating on top of the perovskite layer. However, despite using water-resistive coating, the intrinsic material property makes perovskite chemistries vulnerable to moisture^4,10–12^. Moreover, the use of hydrophobic encapsulation on top of the perovskite layer reduces its light absorption ability and formation of active charge carriers and thus, resulting in reduced photocurrent generation and diminished photovoltaic performance. Use of two-dimensional bilayer or multilayer perovskite architecture such as the incorporation of (C_6_H_5_(CH_2_)_2_NH_3_^+^) into the CH_3_NH_3_PbI_3_ matrix improved moisture stability and allowed ambient fabrication of perovskite solar cells but photovoltaic efficiency and performance were reduced^4^. Similar studies were carried on mixed halide perovskites like CH_3_NH_3_PbI_(3−x)_Br_x_, CH_3_NH_3_PbI_(3−x)_Cl_x_^4^. Increased stability was reported for CH_3_NH_3_PbI_(3−x)_Br_x_ perovskite chemistry where 10–15 mol % I^−^ were replaced with Br^−^. The increase in stability was accredited to the stronger interaction between CH_3_NH_3_^+^ and Br^−^. Furthermore, 2-Aminoethanethiol (2-AET) was reported to be used as a ligand to bridge the organic compound CH_3_NH_3_I (MAI) and inorganic compound PbI_2_ which restricts the decomposition of MAPbI_3_ perovskite structure and prevents fast growth of PbI_2_. The compact (PbI_2_)–2-AET–(MAI) molecule was reported to make the perovskite film intrinsically hydrophobic and the perovskite is reported to retain its crystal structure for > 10 min after immersion in water^10,13^.

Several studies have reported that perovskite film quality depends on the methods of synthesis and materials used for synthesis. The improved film morphology enhances photovoltaic performance and ensures long term stability of the perovskite films. For example, the use of non-halide lead precursors results in the formation of high-quality perovskites, which led to the synthesis of novel perovskite films with improved optoelectronic properties, photovoltaic performance and increased stability^4,10,11^. Similar studies suggested the use of ion-doped or mixed halide perovskites to be an effective solution for improved stability of perovskite material under ambient conditions^4,5^. PSCs fabricated without encapsulation under relative humidity (RH) of 70% using Lead thiocyanate precursor Pb(SCN)_2_ and Lead(II) tetrafluoroborate Pb(BF_4_)_2_, have been reported to show improved moisture resistance as compared to normal PSCs prepared using PbI_2_ precursor. The improved moisture resistance has been attributed to better intrinsic stability of the perovskite material due to the incorporation of SCN^−^ or BF_4_^−^ ions^4^. Furthermore, improved moisture stability was reported on an increasing percentage of (SCN)^−^ and replacing two I^−^ with (SCN)^−^ in CH_3_NH_3_PbI_3_ perovskite composition^4^. However, very limited studies have been performed on a systematic increase of (SCN)^−^ percentage with the corresponding reduction in I^−^ percentage in CH_3_NH_3_PbI_3_ perovskite composition to understand its effect on moisture stability and photovoltaic performance of PSCs.

In this paper, we have applied ab initio modeling to study the optoelectronic properties of thiocyanate doped lead iodide CH_3_NH_3_PbI_(3−x)_(SCN)_x_ perovskite for several compositions with increasing concentration of (SCN)^−^ at x = 0, 0.25, 0.49, 1.0, 1.45 using Vienna Ab initio Simulation Package (VASP). Subsequently, the above-defined compositions were synthesized by varying the concentration of lead thiocyanate (Pb(SCN)_2_) precursor in methylammonium iodide (CH_3_NH_3_I) solution. The synthesized perovskite films with increasing concentration of (SCN)^−^ were characterized by X-ray diffraction (XRD) to identify the perovskite crystal structure, and Raman Spectroscopy to identify the characteristic peaks of the perovskite composition. Photoluminescence spectroscopy and absorption spectroscopy were used to identify the intermediate defect states and bandgap of the material. Further, a comparative analysis was performed on these perovskite chemistries based on the ab initio analysis to rank them for suitability as a photovoltaic candidate and cross-validated the calculated optoelectronic properties with the experimental results. Furthermore, ‘n-i-p’ and ‘p-i-n’ perovskite solar device architectures corresponding to x = 0, 0.25, 0.49, 1.0 thiocyanate doped lead halide perovskite chemistry were fabricated and tested for feasibility, scalability, efficiency and stability for photovoltaic applications. It was found that, among all the fabricated devices with different perovskite chemistries, ‘p-i-n’ PSC device fabricated with CH_3_NH_3_PbI_(3−x)_(SCN)_x_, where x = 1.0 showed the highest stability. The device efficiency was retained until 450 h.

## Methods

### Computational method

Density functional theory calculations were performed on the select CH_3_NH_3_PbI_(3−x)_(SCN)_x_ perovskite stoichiometry with an increasing amount of (SCN)^−^ in steps of x = 0, 0.25, 0.49, 1.0, 1.45. All the calculations were done using VASP software. The valence electronic states were expanded in a basis of plane-waves up to plane-wave kinetic energy of 500 eV. The core-valence interactions were described using the projector augmented wave (PAW) approach^14,15^. The Perdew–Burke–Ernzerhof (PBE) generalized gradient approximation (GGA) functional^4^ was used to describe the electron exchange–correlation interactions. The Brillouin zone was sampled using a gamma centered k-point mesh. The *k*-point grid spacing of 0.2 Å^−1^ was used for reciprocal space integration in structure optimization and spacing of 0.1 Å^−1^ in electronic structure calculations. Geometry relaxation for all the calculations was run until the relaxation forces on atoms were not > 0.01 eV Å^−1^ and the SCF convergence threshold was set to 10^–7^ eV^16^. Complete cell optimization was carried out for all the systems without taking spin orbit coupling into account. Band Structure, Density of States (DOS) and Projected Density of States (PDOS)^17,18^ were plotted using the Xmgrace and Origin software^19,20^.

### Material synthesis

#### Synthesis of electron transport materials (ETMs) and hole transport materials (HTMs)

Titanium dioxide (TiO_2_) and [6,6]-phenyl-C_61_-butyric acid methyl ester (PCBM) were synthesized as ETMs. Spiro-MeOTAD and poly(3,4-ethylenedioxythiophene) polystyrene sulfonate (PEDOTT:PSS) were synthesized as HTMs. A blocking layer of ETM, TiO_2_ was synthesized by addition of 0.02 M HCl (0.62 µl) to 0.2 M titanium Isopropoxide (61 µl) solution in 1 ml ethanol^4^. A mesoporous TiO_2_ layer was synthesized by diluting the TiO_2_ paste in ethanol. PCBM solution acting as ETM was prepared inside the glove box, under the nitrogen (N_2_) environment, by mixing 10 mg PCBM in 1 ml chlorobenzene^21,22^.

Spiro-MeOTAD solution acting as the HTM was synthesized inside the glove box under the N_2_ environment. The synthesis involved the preparation of chlorobenzene and LiTFSI solution^4^. Chlorobenzene solution was formed by dissolving 80 mM (0.196 gm) Spiro-MeOTAD powder and 64 mM (0.0173 g) 4-tert-butylpyridine in 1 ml chlorobenzene. Li-TFSI solution contained 255 mg Li-TFSI in 1 ml acetonitrile. The chlorobenzene solution and 24 mM of Li-TFSI solution were mixed to realize the complete Spiro-MeOTAD solution for HTL fabrication. PEDOT:PSS solution acting as HTM was also prepared inside the glove box under inert conditions by mixing isopropanol and PEDOT:PSS in 3:1 volume ratio and filtered using 0.22 µm Nylon filter^21,22^. Further details on materials, reagents and equipment used for the synthesis of ETMs, HTMs and perovskite chemistries are mentioned in Supplementary Sects. 1 and 2, respectively.

#### Synthesis of MAPbI_3−*x*_ (SCN)_*x*_ perovskite chemistries

Synthesis of MAPbI_3−x_(SCN)_x_ perovskite involved the preparation of separate Lead Iodide (PbI_2_) and Methylammonium Iodide (MAI) solution, inside the glove box under inert conditions at x = 0^4^. PbI_2_ solution was prepared by dissolving 500 mg PbI_2_ powder in 1 ml Dimethylformamide (DMF) solvent, followed by filtration using a 0.22 µm Nylon filter. MAI solution was prepared by dissolving 50 mg MAI powder in 5 ml isopropanol. Synthesis of other MAPbI_3−x_(SCN)_x_ perovskite chemistries were performed under ambient conditions with an increasing percentage of (SCN)^−^. The synthesis involved the preparation of MAI solution and five different Pb(SCN)_2_ solutions with an increasing concentration of Pb(SCN)_2_ powder in the solution. Pb(SCN)_2_ solutions were realized by dissolving Pb(SCN)_2_ powder in concentrations of 500 mg, 550 mg, 650 mg and 750 mg in 1 ml DMF respectively, followed by filtration using a 0.22 µm Nylon filter.

### Device fabrication

**Figure 1:**
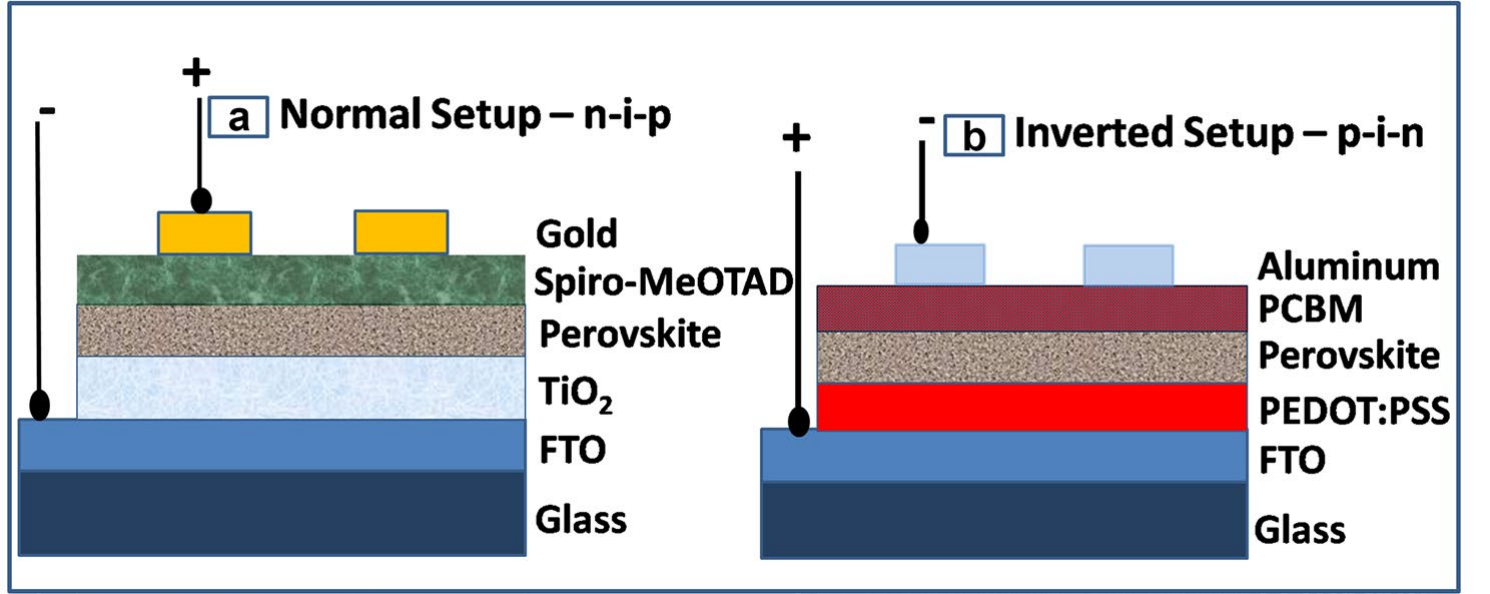
Schematic of (**a**) n-i-p (**b**) p-i-n PSC device architecture. The schematic has been drawn in Microsoft Office PowerPoint.

Two different types of PSC device architecture were realized namely, non-inverted (n-i-p) and inverted (p-i-n). The n-i-p device architecture follows a “bottom-up” approach and light traverses through the transparent FTO glass substrate followed by n-type ETL layer to the active perovskite layer whereas p-i-n device architecture follows a “top-down” approach and light traverses through FTO substrate followed by p-type HTL to active perovskite layer. The incident light as a stream of photons on the surface of the active perovskite material, with greater energy than the bandgap energy of the material, generates electron–hole pairs that are made to flow in opposite direction through ETL and HTL respectively across the external circuit to generate power. The fabricated n-i-p (Fig. 1a) and p-i-n (Fig. 1b) device architectures are as shown in schemes A and B respectively^4,21–32^.

A $${\text{FTO/blocking-TiO}}_{2} /{\text{mesoporous-TiO}}_{2} /{\text{CH}}_{3} {\text{NH}}_{3} {\text{PbI}}_{{(3 - {\text{x}})}} \left( {{\text{SCN}}} \right)_{x} /{\text{Spiro-MeOTAD}}/{\text{Au}}$$

B $${\text{FTO/PEDOT:PSS/ CH}}_{3} {\text{NH}}_{3} {\text{PbI}}_{{(3 - {\text{x}})}} \left( {{\text{SCN}}} \right)_{{\text{x}}} /{\text{PCBM}}/{\text{Ag}}$$

#### Fabrication of n-i-p device architecture

Non-inverted PSCs were fabricated on 2.5 × 2.5 cm^2^ FTO substrates and the fabrication steps are presented in Supplementary Fig. S1^4,23–31^. Multiple cells were fabricated on one Fluorine-doped tin oxide (FTO) plate. Patterned FTO plates were cleaned with neutral liquid detergent and de-ionized (DI) water, followed by bath sonication twice in ethanol for 30 s each and subsequently rinsed twice with DI water for 30 s each. The cleaned FTO substrates were dried under nitrogen and treated with UV-Ozone for 15 min inside a glove box to remove any other contaminants such as photoresists, human skin oil, cleaning solvent residues, etc. from the surface of the substrate.

The UV ozone treated FTO substrates were spin-coated with two layers of TiO_2_ synthesized earlier, a blocking TiO_2_ layer followed by a mesoporous TiO_2_ (mp-TiO_2_) layer to form the complete ETL layer. The blocking TiO_2_ layer was spin-coated at 4,000 r.p.m. for 30 s, followed by sintering at 450° C for 30 min. Subsequently, TiO_2_ paste was spin-coated at 3,000 r.p.m. for 30 s followed by sintering at 500 °C for 30 min^4^. Post sintering, the mp-TiO_2_ layer was treated with 40 mM TiCl_4_ treatment in a beaker of ice-cold DI water at 70 °C for 30 min followed by sintering at 500° C for 30 min^4^. TiCl_4_ treatment is known to substantially reduce surface traps, increase electron injection rate, retard electron–hole recombination and thus improving the overall performance of the photovoltaic device. Thiocyanate doped methylammonium lead iodide active perovskite layer with CH_3_NH_3_PbI_(3−x)_(SCN)_x_ perovskite chemistry was fabricated on top of the mp-TiO_2_ layer by two-step spin-coating procedure at varying Pb(SCN)_2_ concentration. However, at x = 0, filtered PbI_2_ solution was spin-coated on top of the mp-TiO_2_ layer at 3,000 r.p.m. for 40 s and annealed at 90° C for 1 h^4^. Post formation of PbI_2_ film, PbI_2_ coated substrates were dipped in CH_3_NH_3_I solution for 60 s followed by spin coating at 3,000 r.p.m. for 20 s, followed by rinsing with isopropanol and annealing at 80° C for 20 min, to realize CH_3_NH_3_PbI_3_ active perovskite film^4^.

At x > 0, for increased variation of (SCN)^−^ concentration in CH_3_NH_3_PbI_(3−x)_(SCN)_x_ perovskite stoichiometry, perovskite films were realized similarly by two-step spin coating process using varying concentrations of filtered Pb(SCN)_2_ solutions (500 mg, 550 mg, 650 mg Pb(SCN)_2_ powder respectively in 1 ml DMF and CH_3_NH_3_I solution. Spiro-MeOTAD layer was spin-coated on top of perovskite film at 4,000 r.p.m. for 60 s^4^. The Spiro layer being highly moisture-sensitive was spin-coated inside the glove box under N_2_ condition and was stored in the glove box for 48 h before Au deposition. Au electrode of 100 nm thickness was deposited by thermal evaporation on top of the Spiro layer.

#### Fabrication of p-i-n device architecture

Inverted PSCs were fabricated on 2.5 × 2.5 cm^2^ FTO substrates^21,22,32^. FTOs were patterned, cleaned and treated with UV-ozone using a similar procedure as discussed above. The UV ozone treated FTO substrates were spin-coated with the filtered PEDOT:PSS solution at 4,000 r.p.m. for 1 min to form 40–50 nm thick film, followed by annealing at 140 °C for 10 min inside the glove box under N_2_ environment. Fabrication of Thiocyanate doped lead iodide active perovskite layer with CH_3_NH_3_PbI_(3−x)_(SCN)_x_ perovskite chemistry on top of PEDOT:PSS layer for different perovskite compositions was done similarly as discussed for n-i-p devices. PCBM solution was spin-coated on top of the perovskite layer at 1,200 r.p.m. for 40 s to obtain a PCBM ETL layer of approximately 40 nm thickness. Ag metal cathode of 100 nm thickness was finally deposited on the top PCBM layer by thermal evaporation.

## Results

### DFT calculations

Variable cell relaxed structure, band structure, bandgap DOS, PDOS were computed for five different CH_3_NH_3_PbI_(3−x)_(SCN)_x_ stoichiometry at x = 0, 0.25, 0.49, 1.0, 1.45 which corresponds to 0, 8.33, 16.66, 33.32, 49.98% (SCN)^−^ respectively^4^. A 2 × 2 super cell configuration was considered for the calculations such that the material composition at x = 0, 0.25, 0.49, 1.0, 1.45 corresponds to (CH_3_NH_3_)_4_Pb_4_I_12_, (CH_3_NH_3_)_4_Pb_4_I_11_SCN, (CH_3_NH_3_)_4_Pb_4_I_10_(SCN)_2_, (CH_3_NH_3_)_4_Pb_4_I_8_(SCN)_4_, (CH_3_NH_3_)_4_Pb_4_I_6_(SCN)_6_, respectively.

**Figure 2:**
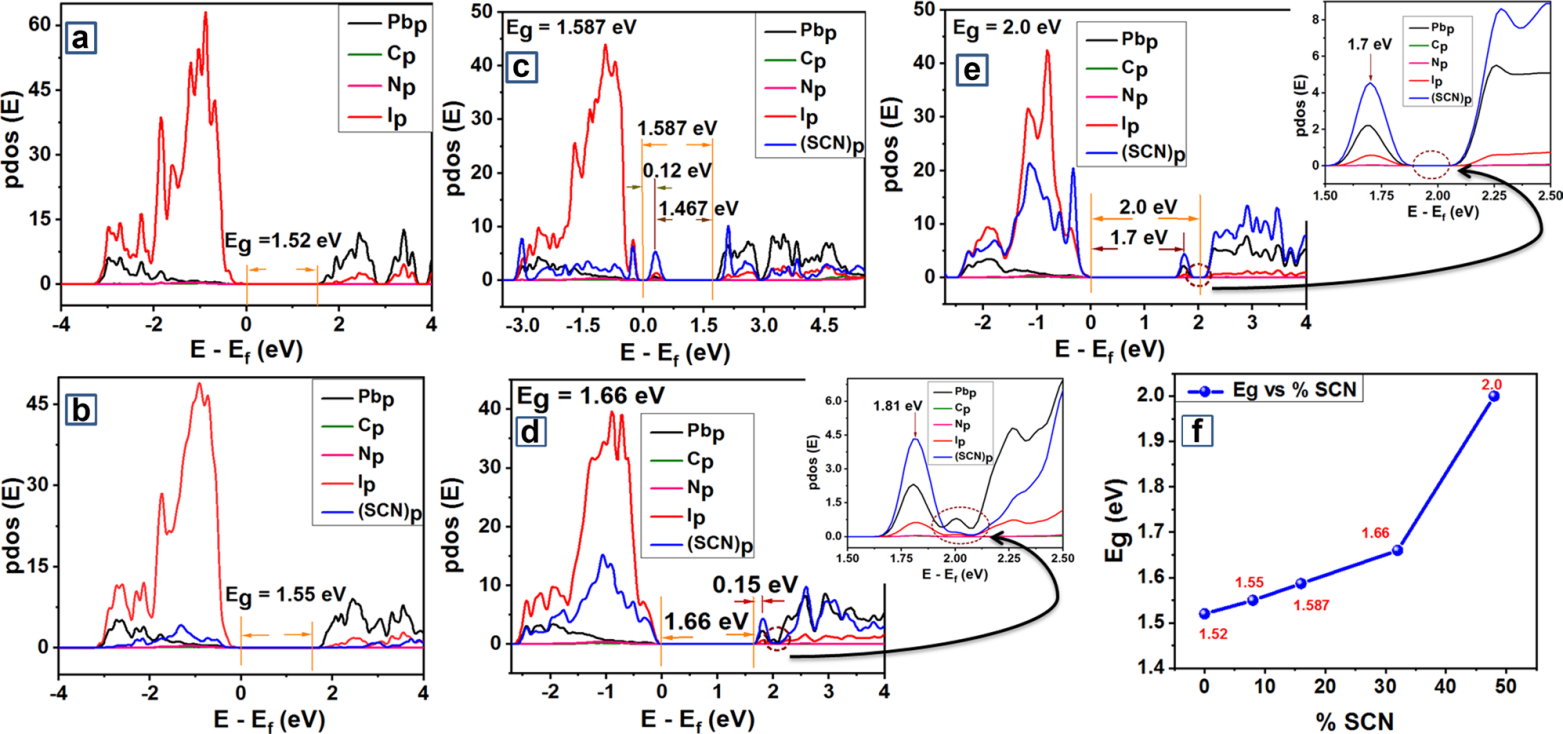
PDOS of CH_3_NH_3_PbI_(3−x)_ (SCN)_x_ perovskite at (**a**) x = 0 (**b**) x = 0.25 (**c**) x = 0.49 (**d**) x = 1.0 (**e**) x = 1.45 . E_f_ represents Fermi energy and all other energies are referenced to 0 of E_f_. (**f**) Variation of Eg versus %SCN. Graphs were plotted using free and open-source software Quantum Espresso (https://www.quantum-espresso.org/, Version: 6.4.1), and ‘gnuplot’ (https://www.gnuplot.info/, Version: 5.2).

The initial atomic structure for these systems was obtained from the optimized structure of (CH_3_NH_3_)_4_Pb_4_I_12_ by replacing appropriate numbers of I^−^ ions with (SCN)^−^ ions. One, two, four, and six I^−^ ions were replaced with (SCN)^−^ to maintain the point group symmetry to obtain the above-mentioned systems respectively. Different configurations were tried by changing the position of (SCN)^−^ in these systems. Structure optimization and relaxation for the different systems followed the same convergence threshold criteria as defined in the Computational methods section. The optoelectronic properties for all the optimized systems were computed and the bandgaps for all perovskite stoichiometry resulted to be “direct” bandgaps. The computed band structure and variable cell optimized crystal structure for all the (SCN)^−^ based systems are shown in Supplementary Figs. S2–S6. Figure 2a–e shows the computed PDOS for these materials with an increase in % (SCN)^−^. All the atomic orbital contributions are summed up to get the overall DOS.

The computed band structure (Supplementary Fig. S2) for (CH_3_NH_3_)_4_Pb_4_I_12_ and (Supplementary Fig. S3) (CH_3_NH_3_)_4_Pb_4_I_11_SCN goes in accordance with the reported structure^4,14^. The computed bandgaps gradually increase with the increase of (SCN)^−^ percentage from 0 to 50 in the perovskite structure (Fig. 2f). The computed bandgaps for perovskite chemistries at x = 0 and x = 0.25 are 1.52 eV and 1.55 eV respectively (Fig. 2a,b). For (CH_3_NH_3_)_4_Pb_4_I_12_ and (CH_3_NH_3_)_4_Pb_4_I_11_SCN top of the valence band is dominated by ‘p’ orbitals of I^−^ and the bottom of the conduction band is dominated by ‘p’ orbitals of (SCN)^−^ (Fig. 2a,b). It is important to note that no intermediate trapped states are observed in the computed band structure of (CH_3_NH_3_)_4_Pb_4_I_11_SCN due to the incorporation of (SCN)^−^, which reduces the chance of carrier recombination process in the perovskite material and thus ensuring longer carrier lifetime. The calculations further indicate strong ionic interactions between (SCN)^−^ and adjacent Pb atoms which should correspond to the improved chemical stability of the perovskite material^4^.

However, an intermediate trapped state appears closer to the valence band at 0.12 eV due to the incorporation of two (SCN)^−^ in the (CH_3_NH_3_)_4_Pb_4_I_10_(SCN)_2_ perovskite structure (Fig. 2c). The computed bandgaps for the corresponding perovskite chemistry at x = 0.49 is 1.587 eV and the energy gap between the intermediate trapped state and conduction band minimum is 1.467 eV (Fig. 2c). The top of the valence band shows hybridization between ‘p’ orbital of (I)^−^ and (SCN)^−^ whereas the bottom of the conduction band is dominated by Pb ‘p’ orbitals. The intermediate defect state is dominated by C ‘p’ orbitals of (SCN)^−^ (Fig. 2c).

In the case of (CH_3_NH_3_)_4_Pb_4_I_8_(SCN)_4_, on the incorporation of four (SCN)^−^ top of the valence band shows a hybridization between I^−^ ‘p’ and (SCN)^−^ ‘p’ orbitals whereas the bottom of the conduction band shows a hybridization between Pb ‘p’ and (SCN)^−^ ‘p’ orbitals (Fig. 2d). The computed bandgap on the incorporation of four (SCN)- in the (CH3NH3)4Pb4I8(SCN)4 structure at x = 1.0 is 1.66 eV as shown in Fig. 2d. One peak appears at 1.81 eV, but it is the continuation of the conduction band and the same is shown by a dotted circle in Fig. 2d. The conduction band minimum was observed at 1.66 eV and the same is marked in Fig. 2d. It is evident from the inset picture of Fig. 2d, that there are occupied states of ‘p’ orbitals of ‘Pb’, ‘C’, and ‘I’ atoms in between the appearing peak and the conduction band. This proves that the peak appearing at 1.81 eV in Fig. 2d lies within the conduction band. Thus, there appear no intermediate defect states in PDOS of perovskite chemistry at x = 1.0 (Fig. 2d), similar to the previous cases at x = 0, 0.25 (Fig. 2b,c) and unlike the perovskite chemistry at x = 0.49.

Further, incorporation of six (SCN)^−^ in the (CH_3_NH_3_)_4_Pb_4_I_6_(SCN)_6_ structure results in a bandgap of 2 eV as shown in Fig. 2e. A peak is also visible at 1.7 eV with low formation energy, closer to the conduction band and the same is depicted in Fig. 2e. However, the peak is not lying within the conduction band and the same is evident from the inset picture of Fig. 2e. This clearly shows that there are no occupiable states in between the appearing peak and the conduction band, thus it implies that the peak appearing at 1.7 eV is an intermediate trapped state. In this case, the top of the valence band shows a hybridization between I^−^ ‘p’ and (SCN)^−^ ‘p’ orbitals, and the bottom of the conduction band shows a hybridization between Pb ‘p’ and (SCN)^−^ ‘p’ orbitals (Fig. 2e). The intermediate defect state is dominated by C ‘p’ orbitals of (SCN)^−^ (Fig. 2e).

It is observed that the bandgap increases with an increase in % (SCN)^−^ (Fig. 2f) and lies between 1.52 and 2 eV. Hence, the computed bandgap for perovskite chemistries at x = 0, 0.25, 0.49, 1.0 falls in the range of > 1.5 eV and < 2 eV which is considered to be the ideal bandgap for a solar material. However, the bandgap of perovskite chemistry at x = 1.45 is 2 eV which is not preferred for photovoltaic applications. Also, perovskite stoichiometry simulated at x = 0.49 (Fig. 2c) and 1.45 (Fig. 2e) have intermediate trapped states which can result in deterioration of photovoltaic performance and hence may not be a good choice for the fabrication of solar cells. Further, an increase in SCN percentage has been reported beneficial for improved film morphology, intrinsic stability, moisture resistance, and long term ambient stability^4,33^.

Therefore, with intermediate defect states at x = 0.49, 1.45, and non-favorable bandgap at x = 1.45, the perovskite chemistries at x = 0.49 and 1.45 with 16.66% and 50% (SCN)^−^ should not be considered as a viable candidate for the fabrication of perovskite solar cells. However, perovskite chemistries at x = 0, 0.25, 1.0 are viable for the fabrication of solar cells because of their favorable bandgap and absence of an intermediate trapped state. But, CH_3_NH_3_PbI_3−x_(SCN)_x_ perovskite simulated at x = 1.0 with 33.32% (SCN)^−^ has the highest percentage of (SCN)^−^ among the perovskite chemistries at x = 0, 0.25, 1.0. Therefore, considering the criteria of better moisture resistance at a higher percentage of (SCN)^−^, perovskite chemistry at x = 1.0 with favorable bandgap and no intermediate defect states can be considered as the best viable candidate for photovoltaic applications.

### Characterization of perovskite materials

The synthesized CH_3_NH_3_PbI_3−x_(SCN)_x_ perovskite stoichiometry was characterized by XRD and Raman Spectroscopy to confirm the formation of the perovskite material. Further characterizations were done by UV–Vis absorption spectroscopy and Photoluminescence spectroscopy (PL) to find the optical bandgap of the synthesized perovskite materials and estimate the presence of localized defect states in the material between the valence and conduction band respectively. DFT computed band structure and PDOS were validated against experimental results.

#### XRD and Raman spectroscopy

The XRD pattern and Raman spectra for CH_3_NH_3_PbI_3−x_(SCN)_x_ synthesized with 650 mg/ml Pb(SCN)_2_ solution which confirms the formation of the material are presented in Supplementary Fig. S7 and Supplementary Sect. 3.

#### UV–Vis spectroscopy

**Figure 3:**
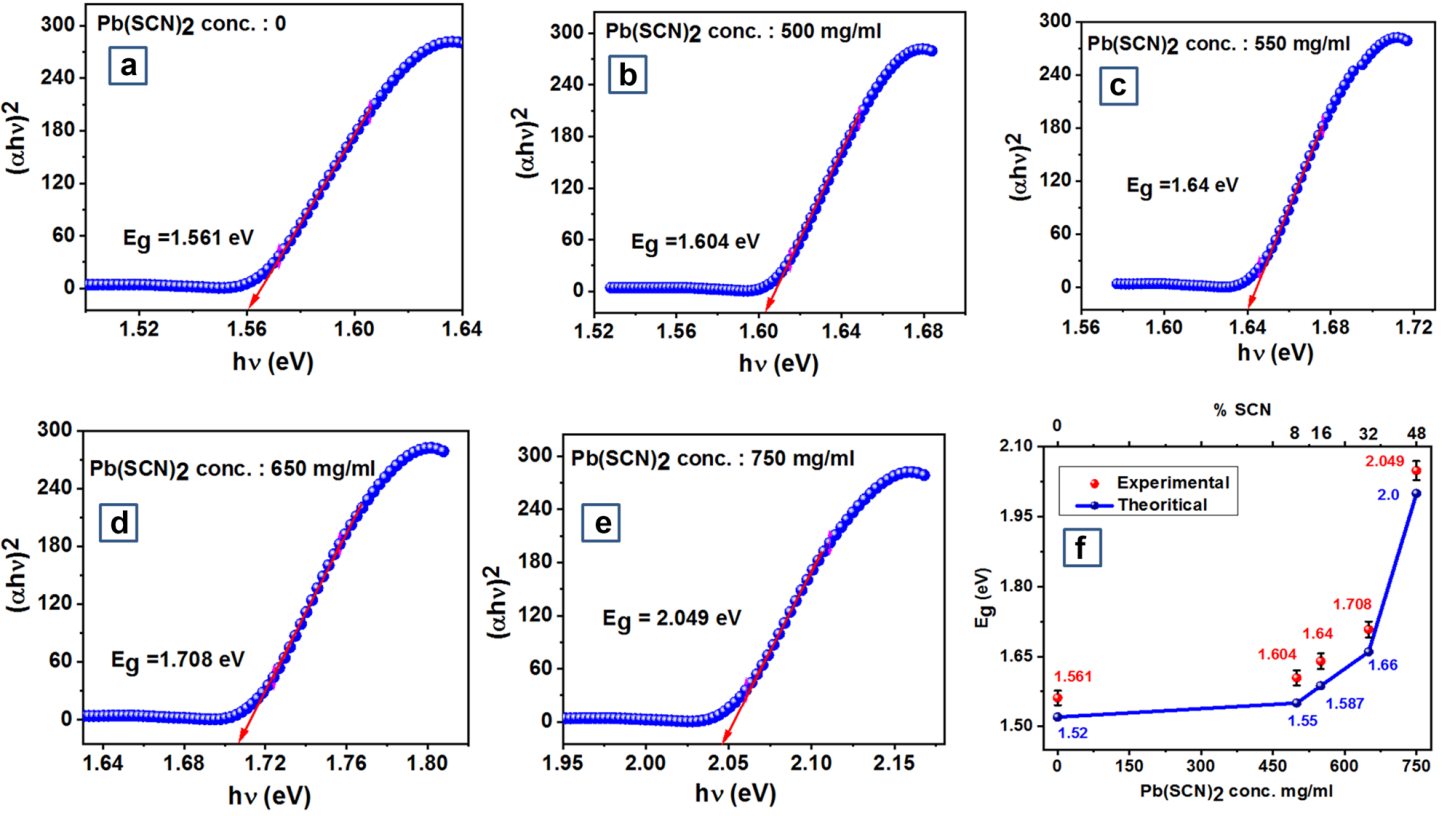
Tauc plot for the synthesized chemistries at varying Pb(SCN)_2_ concentration (**a**) 0 (**b**) 500 mg (**c**) 550 mg (**d**) 650 mg (**e**) 750 mg (**f**) Correlation of experimental bandgap versus DFT computed bandgap for different perovskite stoichiometry. Graphs were plotted using free and open-source software ‘gnuplot’ (https://www.gnuplot.info/, Version: 5.2).

Figure 3a–e presents the Tauc plots and calculated bandgap obtained from UV–visible absorption spectra for various compositions of CH_3_NH_3_PbI_(3−x)_(SCN)_x_ perovskite stoichiometry at x = 0 and x > 0. Absorbance is measured using a UV–vis spectrometer as a function of the wavelengths on passing light within the wavelength range of 200–1,200 nm, through the sample. For realizing high-efficiency solar cells, most of the absorption must be in the visible spectrum and very less in the UV and IR regions.

UV absorption portrays absorption coefficients (α) at a different visible wavelength (λ in nm) and the optical bandgap of the material is calculated by plotting Tauc plot from UV absorption spectra results. The Tauc plot is plotted for (αhν)^2^ versus (hν) where ‘hν’ corresponds to the bandgap energy of the material. The resulting Tauc plot has a distinct linear regime which denotes the onset of absorption. Thus, extrapolating this linear region to the abscissa yields the energy of the optical bandgap of the material as shown in Fig. 3a–e. According to the Tauc plot, the calculated optical bandgap gradually increases with an increase in the concentration of Pb(SCN)_2_ in the CH_3_NH_3_PbI_(3−x)_(SCN)_x_ perovskite structure (Fig. 3f). For the five different perovskite systems, we have synthesized, the calculated bandgap from the Tauc plot is very close to the DFT computed bandgaps and hence enables in establishing a correlation between material bandgap (Eg), Pb(SCN)_2_ concentration, and (SCN)^−^ percentage in the synthesized perovskite systems and the same is presented in Fig. 3f. Even though the bandgap computed via the PBE-GGA approach underestimated the experimental bandgap, the difference margin was less as spin orbit coupling (SOC) was not considered in our theoretical calculations. It has been reported that using GGA functional for computing bandgaps of methylammonium lead halide type perovskite systems, exclusion of SOC reduces the difference between experimental and DFT calculated bandgap^4,34^. As depicted by the Tauc plot in Fig. 3e, the bandgap (Eg) for the synthesized material with 750 mg/ml Pb(SCN)_2_ concentration is greater than 2 eV (2.049 eV) and hence it may not be a viable solar material for fabrication of PSCs. For the other 4 perovskite chemistries bandgap falls in the range of ideal solar material and hence can be considered as an active material for PSC fabrication.

### PL spectroscopy

**Figure 4:**
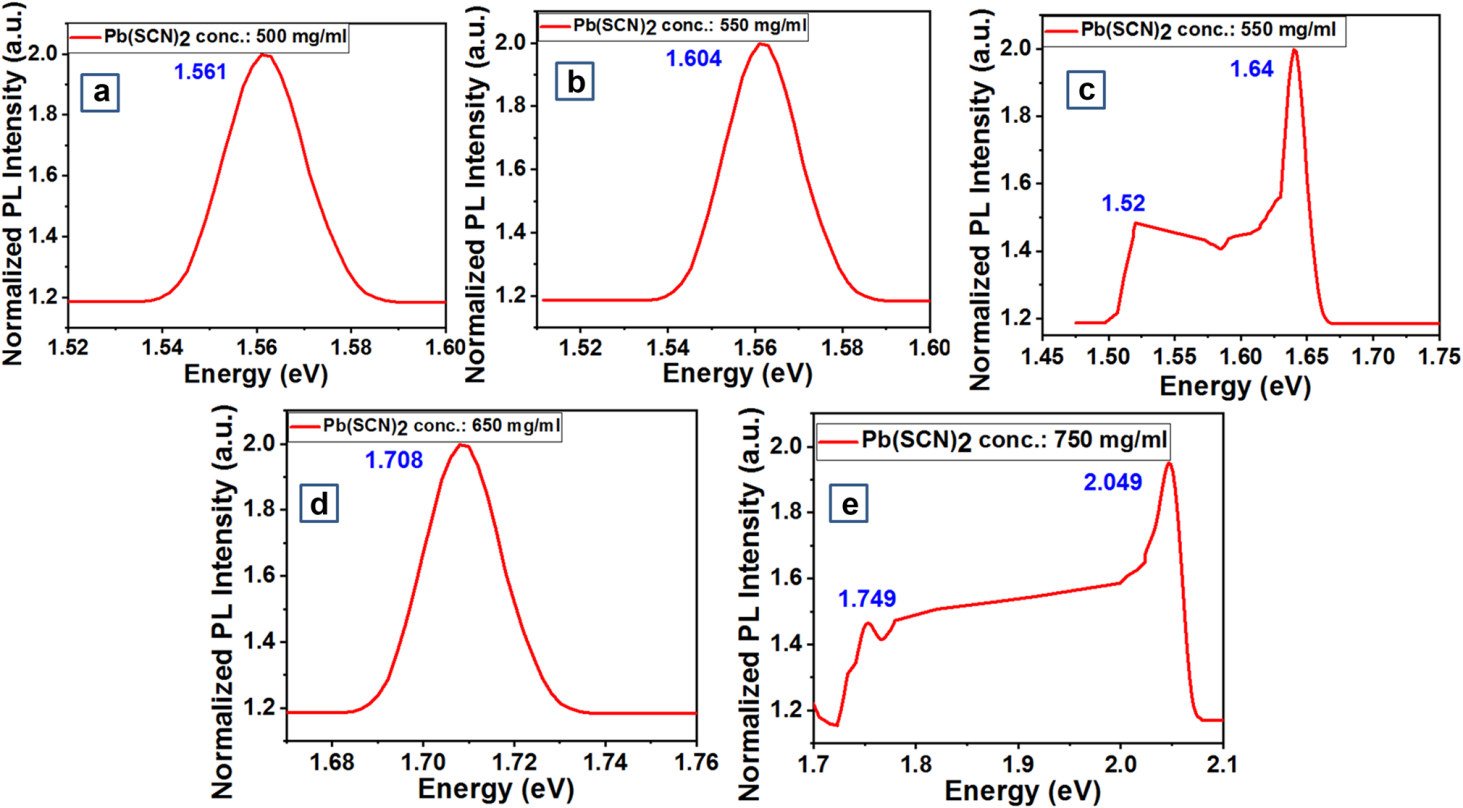
PL spectra for the synthesized chemistries at varying Pb(SCN)_2_ concentration (**a**) 0 (**b**) 500 mg (**c**) 550 mg (**d**) 650 mg (**e**) 750 mg. Graphs were plotted using free and open-source software ‘gnuplot’ (https://www.gnuplot.info/, Version: 5.2).

Figure 4a–e presents the PL for the 5 perovskite stoichiometry at x = 0, 0.25, 0.49, 1.0, 1.45 with increased Pb(SCN)_2_ concentration (mg/ml) in steps of 0, 500, 550, 650, respectively. The obtained PL spectra (Fig. 4a–e) can be well correlated to the computed PDOS via DFT calculations implying correct synthesis of the DFT computed materials (Fig. 2a–e). PL spectra portray the emission peaks corresponding to the localized defect states or shallow levels present in the electronic band structure of the synthesized material.

Some of the PL spectra (Fig. 4a,b,d) show a single emission peak which implies there are no intermediate defect states and corresponds to the bandgap of the material and the results can be compared with the computed PDOS for perovskite chemistries at x = 0, 0.25, 1 respectively (Fig. 2a,b,d). PL spectra for perovskite chemistry at x = 0.49 and 1.45 show two emission peaks at 1.52, 1.64 and 1.749, 2.049 eV, respectively (Fig. 4c,e). 1.64 eV and 2.049 correspond to the bandgap of the material at x = 0.49, 1.45 respectively. However, the other emission peaks at 1.52 and 1.749 eV (Fig. 4c,e) correspond to the presence of intermediate defect states similar to computed PDOS (Fig. 4c,e) between the top of the valence band and bottom of the conduction band for the perovskite stoichiometry at x = 0.49,1 respectively.

Moreover, according to PL spectra for perovskite chemistry at x = 0.49, 1.45 the gap between the two emission peaks is 0.12 eV (Fig. 4c) and 0.3 eV (Fig. 4e). This gap is similar to the computed gap between the two peaks [1.587 eV, 1.467 eV] and [1.7 eV, 2 eV] respectively as projected in PDOS (Fig. 2c,e) implying correct synthesis of the DFT computed materials. The bandgap obtained from the PL for these 5 different perovskite chemistries (Fig. 4a–e) goes in accordance with the bandgap calculated from the Tauc plot (Fig. 3a–e). Hence, post material synthesis and characterization we could conclude the perovskite chemistry at x = 1.0 should be the best viable solar material for PSC fabrication in terms of favorable material bandgap, absence of intermediate defect states and 33.32% (SCN)^−^ in the perovskite chemistry. However, n-i-p and p-i-n PSC devices were fabricated with all perovskite chemistries and were validated for the best solar material in terms of stability.

### Characterization of perovskite solar cell

As mentioned in previous sections, n-i-p and p-i-n PSC devices using the varying composition of CH_3_NH_3_PbI_(3−x)_(SCN)_x_ (at x = 0, 0.25, 0.49, 1) perovskite stoichiometry were fabricated on FTO substrates. However, we could not fabricate efficient PSC devices at x = 1.45 (i.e. 49.98% (SCN)^−^ doping) in the perovskite chemistry as the Pb(SCN)_2_ solution becomes highly viscous during material synthesis resulting in non-uniform film morphology and reducing the efficiency of the fabricated devices. The device characterizations were performed on perovskite solar cells kept in ambient conditions without encapsulation, for all n-i-p and p-i-n device architectures. However, it was found that among all the n-i-p and p-i-n devices fabricated, devices with CH_3_NH_3_PbI_(3−x)_(SCN)_x_ perovskite at x = 1.0 showed the highest stability with moderate efficiency. However, n-i-p devices fabricated at x = 1.0 showed higher efficiency and lesser stability as compared to the fabricated p-i-n devices at x = 1.0. For fabricated p-i-n devices at x = 1.0, the device efficiency was retained until 450 h. The n-i-p and p-i-n devices were fabricated according to the architectures mentioned in Fig. 1a,b. The detailed characterization of the n-i-p and p-i-n device architectures fabricated with perovskite chemistry CH_3_NH_3_PbI_(3−x)_(SCN)_x_ at x = 1.0 are mentioned below.

#### Dark characteristics

Dark characteristics were performed on the fabricated devices at x = 1.0 for n-i-p and p-i-n device architectures to calculate the barrier potential (Vbi), diode ideality factor (n), and diode saturation currents (Io) are presented in Fig. 7a–d.

Using the diode current equation (Eq. 1) and taking the natural log of both sides (Eq. 2), ‘n’, and ‘Io’ are calculated across the diode where ‘Vd’ is the diode voltage.

1 $${\text{ID}} = {\text{Io}}\left[ {e^{{\left[ {\frac{qVbi}{{nKT}}} \right]}} - 1} \right]$$

2 $$\ln {\text{ I}}_{{\text{D}}} = \frac{qVbi}{{nKT}} + \ln {\text{Io}}$$

**Figure 5:**
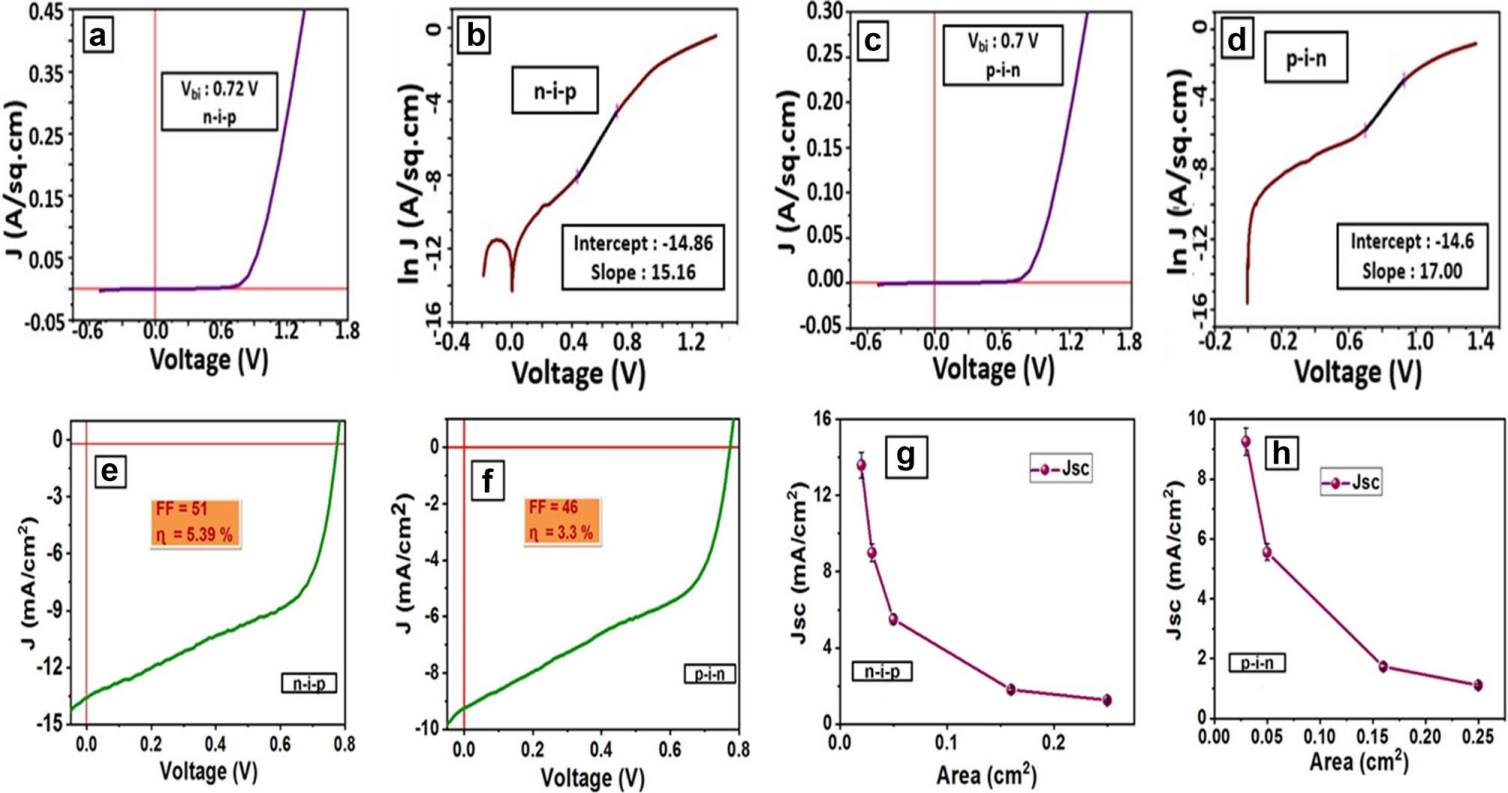
Dark and photovoltaic characteristics and scalability of PSC using perovskite chemistry at x = 1.0 (**a**) Dark ‘n-i-p’ J-V (**b**) Dark ‘n-i-p’ ln J-V (**c**) Dark ‘p-i-n’ J-V (**d**) Dark ‘p-i-n’ ln J-V (**e**) Photovoltaic n-i-p’ J-V (**f**) Photovoltaic ‘p-i-n’ J-V (**g**) Scalability ‘n-i-p’ Jsc versus Area (**h**) Scalability ‘p-i-n’ Jsc versus Area. Graphs were plotted using free and open-source software ‘gnuplot’ (https://www.gnuplot.info/, Version: 5.2).

The gradient and Y-intercept of the linear plot (Eq. 4) allow estimation of ideality factor and Io respectively. The calculated ‘Vbi’, ‘Io’, ‘n’ are 0.72 V, 3.51 × 10^–7^ A , 2.54 (Fig. 5a,b) and 0.7 V, 4.56 × 10^–7^ A , 2.26 (Fig. 5c,d) for n-i-p and p-i-n devices at respectively. Higher Vbi contributes to higher open-circuit voltage (Voc) and thus helps in the improved photovoltaic performance of the PSC. Deviations in the ideality factor from one indicate that, either there are unusual recombination mechanisms taking place or that the recombination is changing in magnitude. Higher is the recombination in the device, lower will be the short circuit current of the solar cell, and lower current results in improved perovskite film morphology.

#### Photovoltaic characteristics and device scalability

Photovoltaic characteristics under a light intensity of 1,000 W/m^2^ n-i-p (Fig. 5e) and p-i-n (Fig. 5f) perovskite stoichiometry at x = 1.0 are presented in Fig. 5e,f. According to J-V characteristics n-i-p device produces an efficiency of 5.39% with a Fill Factor (FF) of 51, Voc of 0.77 V, and short circuit current density (Jsc) of 13.63 mA/cm^2^ (Fig. 5e). However, efficiency produced by p-i-n device at a FF of 46 and Voc of 0.78 V is 3.39% which is less compared to the n-i-p devices due to lower Jsc of 9.34 mA/cm^2^ (Fig. 5f). Device fabrication has been done varying the active device area to check on the scalable efficiency of the device. Unfortunately, with the increase in device area, Jsc decreases resulting in a decrease of PSC power conversion efficiency for both n-i-p and p-i-n device architecture (Fig. 5g,h)

#### Dependency of solar parameters on light intensity

**Figure 6:**
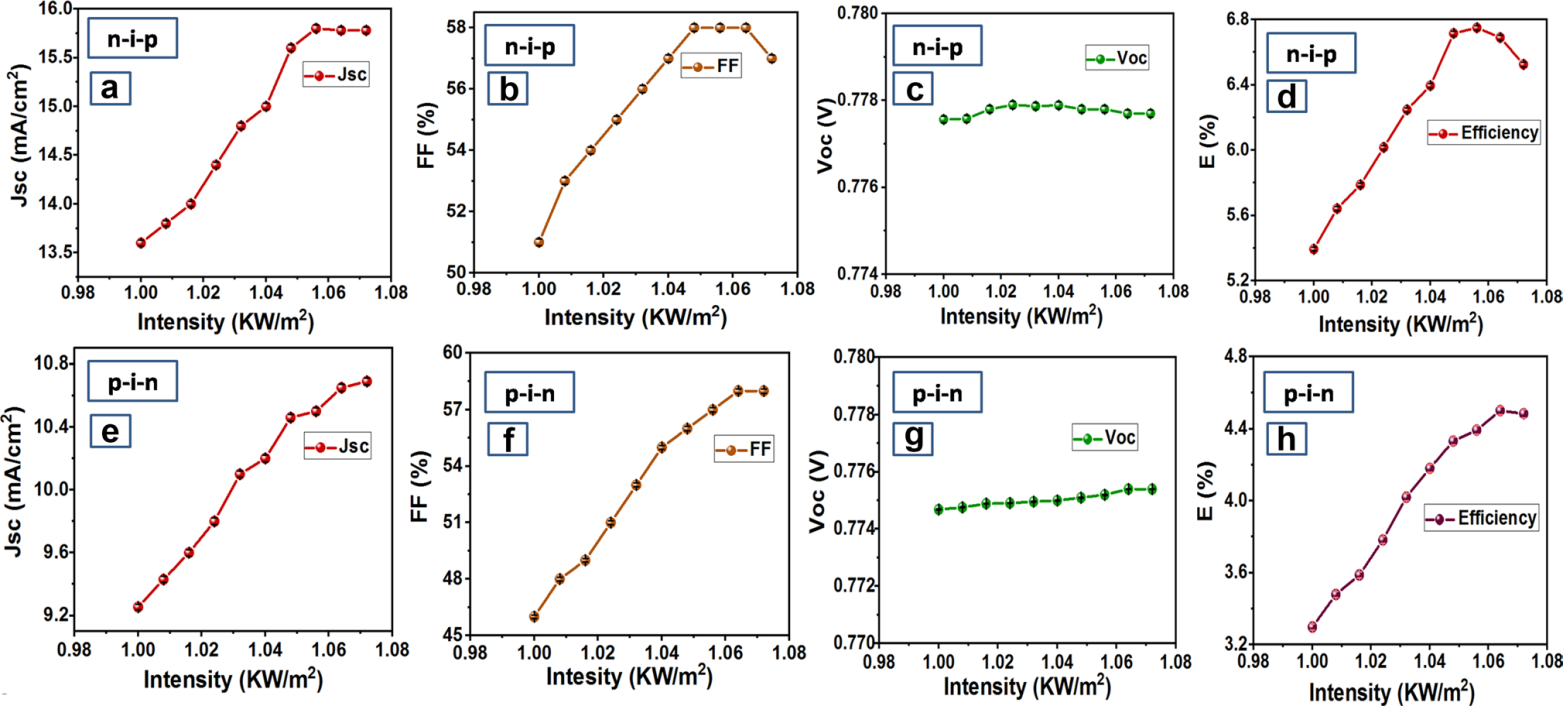
Parameter variation w.r.t. light intensity for PSCs fabricated with perovskite chemistry at x = 1.0 (**a**) ‘n-i-p’ Jsc versus intensity (**b**) ‘n-i-p’ FF versus intensity (**c**) ‘n-i-p’ Voc versus intensity (**d**) ‘n-i-p’ efficiency versus intensity (**e**) ‘p-i-n’ Jsc versus intensity (**f**) ‘p-i-n’ FF versus intensity (**g**) ‘p-i-n’ Voc versus intensity (**h**) ‘p-i-n’ efficiency versus intensity. Graphs were plotted using free and open-source software ‘gnuplot’ (https://www.gnuplot.info/, Version: 5.2).

On increasing light intensity considerable changes were observed in the n-i-p and p-i-n solar cell characterization parameters such as Voc, Jsc, FF, and Efficiency (Fig. 6a–h). An increase in light intensity from 1 solar, results in a gradual increase of Jsc in the case of n-i-p (Fig. 6a) and p-i-n devices (Fig. 6e) respectively. However, Jsc saturates beyond 1.06 kW/m^2^ light intensity in the n-i-p device, unlike p-i-n device which implies the chosen material is capable of absorbing more solar energy. FF increases to its peak up to a certain light intensity and then gradually decreases for n-i-p device (Fig. 6b) and saturates for p-i-n device (Fig. 6f) presumably due to the degradation of the top electrode. Voc almost remains constant with the increase in light intensity for both n-i-p (Fig. 6c) and p-i-n (Fig. 6g) resulting in better device stability. Changes in Jsc, FF, Voc results in an increase of efficiency up to a certain light intensity and then decreases owing to the decrease in FF in n-i-p device and saturates owing to the saturation of FF in p-i-n device (Fig. 6e–h). The saturation of efficiency for p-i-n device indicates better stability of the p-i-n device as compared to the n-i-p device.

### Stability analysis of solar cells

**Figure 7:**
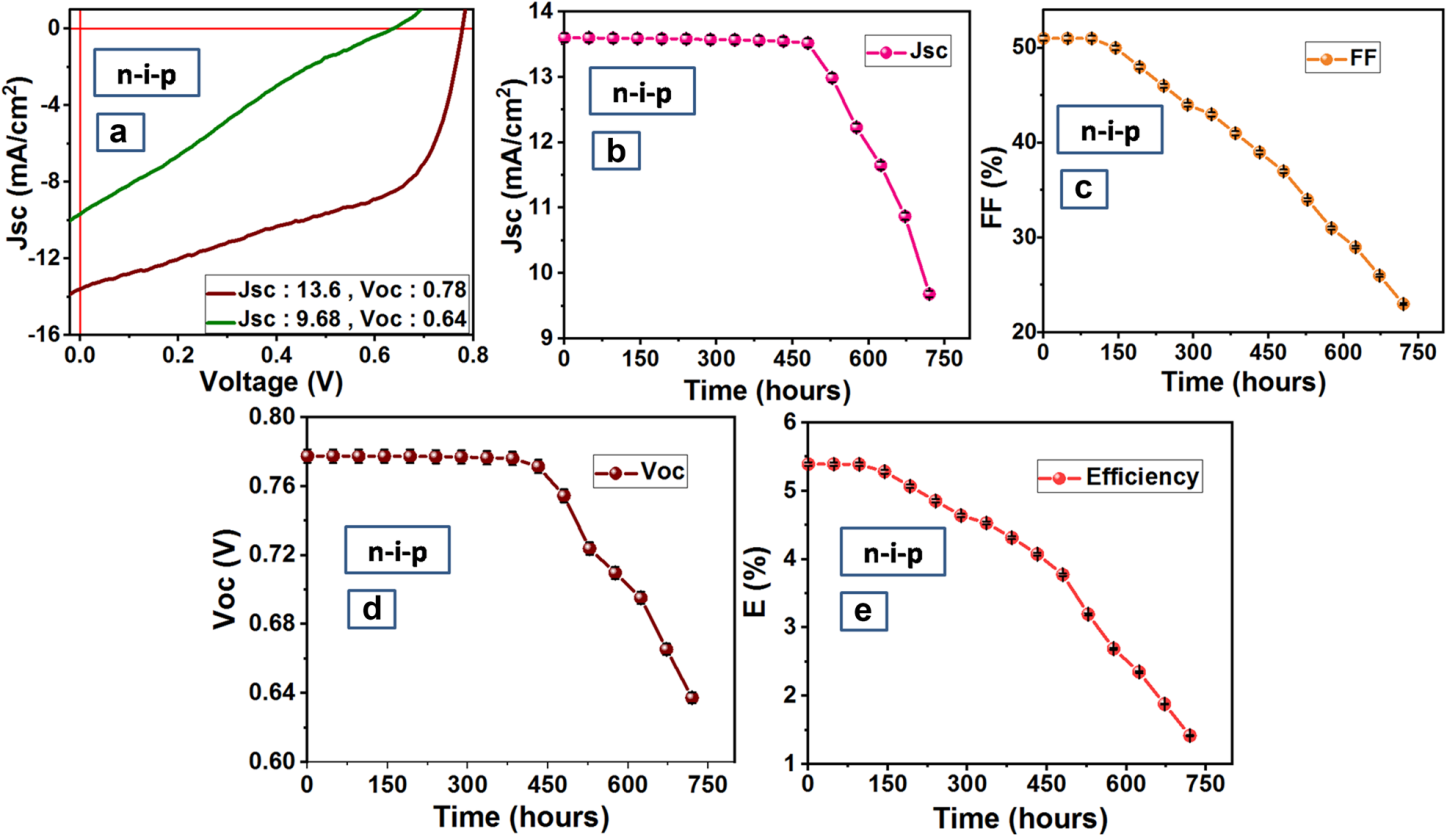
Stability analysis of ‘n-i-p’ PSC fabricated with perovskite at x = 1.0 (**a**) Variation of Jsc w.r.t. V for 750 h (**b**) Jsc versus time (**c**) FF versus time (**d**) Voc versus time (**e**) E versus time. Graphs were plotted using free and open-source software ‘gnuplot’ (https://www.gnuplot.info/, Version: 5.2).

**Figure 8:**
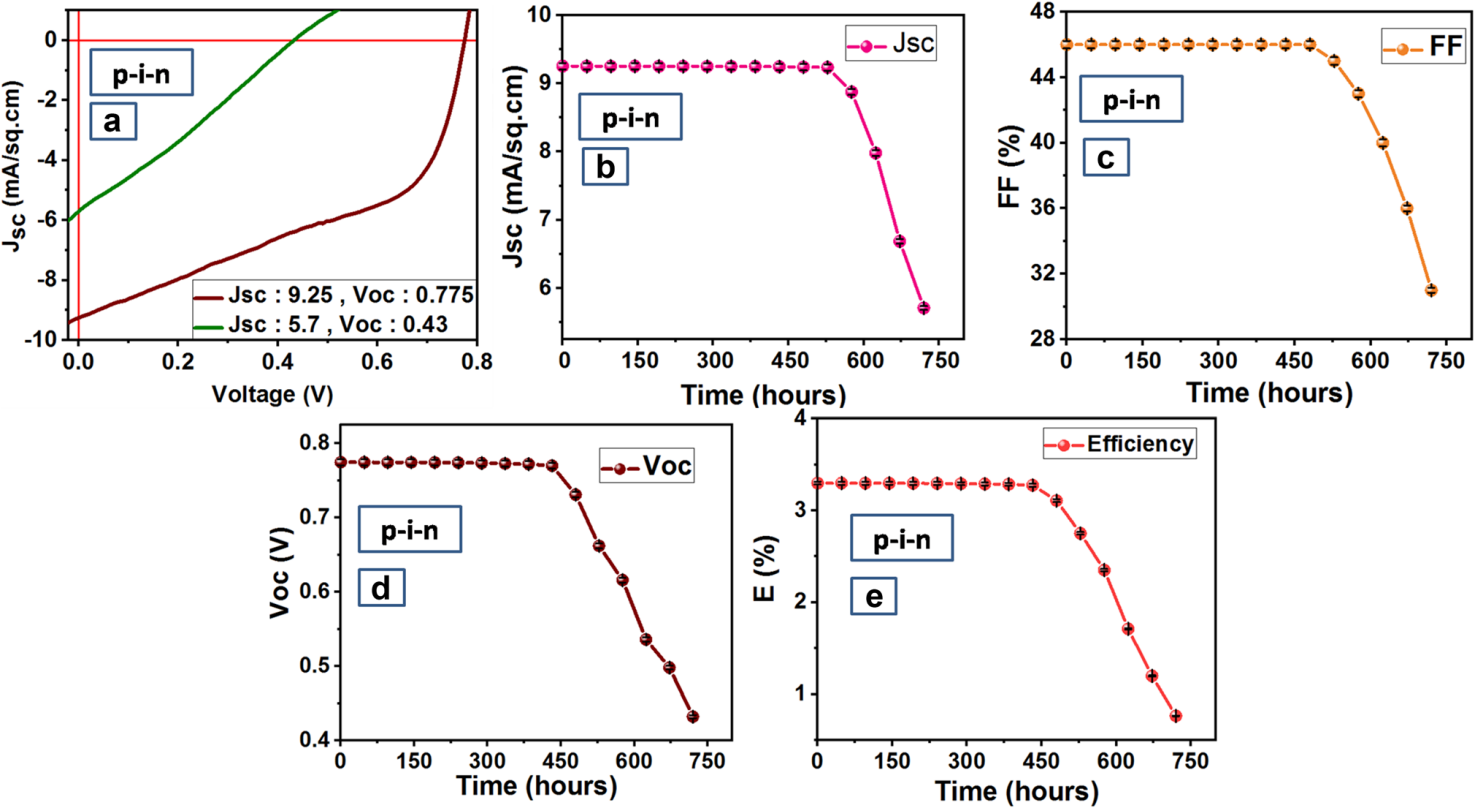
Stability analysis of ‘p-i-n’ PSC fabricated with perovskite at x = 1.0 (**a**) Variation of Jsc w.r.t. V for 750 h (**b**) Jsc versus time (**c**) FF versus time (**d**) Voc versus time (**e**) E versus time. Graphs were plotted using free and open-source software ‘gnuplot’ (https://www.gnuplot.info/, Version: 5.2).

Exposure to ambient conditions and increased recombination at the interfaces results in degradation and reduction of efficiency of perovskite solar cells over time. This efficiency degradation can be attributed to the reduction in solar cell parameters Jsc, FF, Voc over time. Figures 7a–e and 8a–e present the variation in J–V curve, Jsc, FF, Voc, and Efficiency over 750 h for n-i-p and p-i-n devices respectively.

As shown in Figs. 7a and 8a, Jsc dropped down from 13.6 to 9.68 mA/cm^2^ for n-i-p (Fig. 7a) and from 9.25 mA/cm^2^ to 5.7 mA/cm^2^ for p-i-n (Fig. 8a) device after 750 h. Jsc remains constant up to 450 and 525 h for n-i-p (Fig. 7b) and p-i-n (Fig. 8b) devices respectively beyond which Jsc drops down. However, unlike consistency in Jsc, FF drops after 150 h in the case of the n-i-p device (Fig. 7c) presumably due to the degradation of the top Au electrode. But the scenario is different in the case of p-i-n device and FF remains stable for 525 h (Fig. 8c) which contributes to better stability of p-i-n device. Voc was found to remain stable for 450 h both in the case of n-i-p and p-i-n device (Figs. 7d, 8d). Since the efficiency of a PSC device is directly proportional to parameters Jsc, FF, and Voc, despite stability in Jsc and Voc for 450 h, a fast decrease in FF causes a rapid decrease in efficiency of n-i-p device after 150 h (Fig. 7e). Hence in the case of n-i-p device, the variation of efficiency over time follows the same trend as FF. However, in the case of the fabricated p-i-n device, Jsc and FF remain stable up to 525 h and Voc up to 450 h and thus efficiency also remains stable for a longer time of 450 h (Fig. 8e). Hence in the case of the p-i-n device, the variation of efficiency over time follows the trend of Voc.

### Fabrication and characterization of solar panel

A solar panel was made on acrylic with an array of solar cells connected as a combination of series and parallel. The panel consists of 12 FTO pieces as depicted in Supplementary Fig. S8. Each FTO piece consists of 3 p-i-n PSC devices fabricated with perovskite at x = 1.0 as an active light-harvesting material. The p-i-n solar cells provided longer stability with moderate efficiency and hence were used for the formation of a solar panel. Some of the PSCs were connected in series and some in parallel to get an increased Voc and Jsc respectively. The panel consists of three modules with four FTO pieces in each module connected in parallel and the three modules connected in series. On an average, each PSC generates a Jsc of 9 mA/cm^2^ and a Voc of 0.75 V. The overall solar panel generated a Jsc of 108 mA/cm^2^ and Voc of 2.25 V. The overall solar cell efficiency decreases to 3%. However, the efficiency was retained for 5,000 h.

## Discussion

We have successfully computed the electronic properties such as band structure, bandgap, DOS, and PSDOS using DFT calculation for different perovskite CH_3_NH_3_PbI_(3−x)_(SCN)_x_ stoichiometry at x = 0, 0.25, 0.49, 1.0, 1.45 in ambient condition for varying (SCN)^−^ concentrations. DFT calculations were followed by material synthesis and characterization of different perovskite chemistries. DFT approach was used to understand the optimized structure for varying (SCN)^−^ concentration. Further, a correlation was established between DFT computed perovskite stoichiometry at a varying percentage of (SCN)^−^ and synthesized perovskite chemistries at varying concentrations of Pb(SCN)_2_. The emission peaks observed in the DFT computed PDOS resembled the emission peaks found in the PL spectra of synthesized materials, thus confirming the correct synthesis of DFT computed materials. CH_3_NH_3_PbI_(3−x)_(SCN)_x_ perovskite at x = 1.0 was considered as a best viable candidate for solar material for fabrication of PSC devices because of ideal optical bandgap suitable for solar applications, absence of intermediate defect states and a higher concentration of (SCN)^−^. However, PSC devices were fabricated and characterized with all five different perovskite stoichiometry for two different architectures namely n-i-p and p-i-n. Post fabrication and characterization, it was found that n-i-p PSC devices fabricated with CH_3_NH_3_PbI_(3−x)_(SCN)_x_ perovskite at x = 1.0 showed the highest stability with moderate efficiency when exposed to ambient conditions, as compared to the PSC devices fabricated with other perovskite stoichiometry. Thus it can be concluded that the material ranked as the best solar material as per DFT simulations resulted in the fabrication of most moisture-stable perovskite solar cells. Further, it was concluded that between n-i-p and p-i-n PSC devices fabricated with CH_3_NH_3_PbI_(3−x)_(SCN)_x_ perovskite at x = 1.0, p-i-n PSC device showed the highest stability and efficiency up to 450 h under ambient conditions without encapsulation.

## Supplementary information

Supplementary information.
