# Supplementary material for: An inherent instability study using ab initio computational methods and experimental validation of Pb(SCN)2 based perovskites for solar cell applications
Source: Sci Rep. 2020 Sep 17;10:15241. doi: 10.1038/s41598-020-72210-4 (PMC7498586; doi:10.1038/s41598-020-72210-4)
Supplement: Supplementary file 1 — Supplementary information. [file 41598_2020_72210_MOESM1_ESM.pdf]

Supplementary Information

Manuscript Type: Original Research

An inherent instability study using ab-initio computational methods and experimental validation of $Pb(SCN)_2$ based perovskites for solar cell applications

Jayita Dutta[1], Mithun Chennamkulam Ajith[2], Soumya Dutta[2], Umesh R. Kadhane[3], Jinesh Kochupurackal B[3], Beena Rai[1]

[1]Physical Sciences Research Area, Tata Research Development and Design Centre, TCS Research, Tata Consultancy Services, 54, B Hadapsar Industrial Estate, Pune, 411013, India. [2]Indian Institute of Technology Madras, Chennai, Tamil Nadu, 600036, India. [3]Indian Institute of Space Science and Technology, Valiamala Road, Valiamala, Trivandrum, Kerala, 695547, India. Correspondence and requests for materials should be addressed to J.D. (email: jayita.dutta@tcs.com/ jayita.ece2013@gmail.com

1. Materials and Reagents

FTO glass substrates (resistivity 7 Ω/sq.cm) and zinc dust (particle size < 10 µm, assay >= 98%) for substrate patterning were used for patterning the bottom electrodes on FTO coated glass substrates. Titanium isopropoxide (99.999% trace metal basis), conc. HCl (37%), ethanol(denatured with about 1% methyl ethyl ketone for analysis EMSURE®), $TiCl_4$ solution (0.09 M in 20% HCl) and transparent blocking layer of Titanium dioxide (TiO_2) paste were used to synthesize and fabricate the Electron transportation layer (ETL) for 'n-i-p' PSC device architectures. $Pb(SCN)_2$ powder (99.5% trace metal basis), PbI_2 powder (99.999% trace metals basis, perovskite grade), N N-Dimethylformamide (DMF, 99%), CH_3NH_3I powder (98%), isopropanol (>=99.5%) and Nylon filter (0.22 µm) were used to synthesize and fabricate the active

perovskite layer for ‘n-i-p’ and ‘p-i-n’ PSC device architectures. Spiro-MeOTAD powder (99%, HPLC), chlorobenzene (anhydrous, 99.8%), acetonitrile (anhydrous, 99.8%), 4-tert-butylpyridine (98%) and Li-TFSI (>= 99%) were used to synthesize and fabricate the Hole transportation layer (HTL) for ‘n-i-p’ PSC device architectures. Poly(3,4-ethylenedioxythiophene)-poly(styrenesulfonate) i.e. PEDOT:PSS (1.3 wt % dispersion in H_2O, conductive grade), Nylon filter (pore size 0.22 µm, diam. 47 mm) and isopropanol (>=99.5%) were used to prepare the HTL for ‘p-i-n’ PSC device architectures. Chlorobenzene (anhydrous, 99.8%) and Phenyl-C61-butyric acid methyl ester (PCBM) were used to prepare the ETL for ‘p-i-n’ PSC device architectures. Gold wire and Silver wire were used as the top electrode in n-i-p and p-i-n device architectures, respectively. TiO_2 paste was purchased from Solaronix and all other materials and reagents were purchased from Sigma Aldrich. The materials were used directly in the condition as received from Sigma Aldrich and Solaronix.

2. Instruments

Substrate patterning, cleaning and bath sonication (Elma Bath Sonicator, Model: Elmasonic P60H) were prepared in Wet Bench (HEPA Filter equipped). Measurements of all reagents for material synthesis were performed using Measuring Balance (Mettler Toledo). Fabrication of ETL, HTL and Perovskite layer of PSC device were performed using Holmarc Spin Coater (Model No: HO-TH-05). Synthesis of Spiro-MeOTAD HTL layer was performed inside glove box (Mbraun) under N_2 environment (2 to 5 psi pressure) and fabrication was performed inside glove box using POLOS SPS spin coater. Absorption Spectrophotometer (Cary 100 UV-Vis, Agilent), Photoluminescence Spectroflurometer (FluroMax-4, HORIBA), X-ray Diffractometer (Bruker D8) and Raman Spectrometer (inVia Raman Microscope, Renishaw), were used for optoelectronic and structural characterization of synthesized perovskite chemistries. The J–V curves measurement, stability

analysis and all other PSC device characterization were performed under AM 1.5G-simulated illumination using Solar Simulator (Photo Emission Tech, Model No: CT50AAA) with a power density of 1000 W/m^2.The light intensity was calibrated using a certified NREL Standard Cell.

3. XRD & Raman Spectroscopy

The XRD pattern for $CH_3NH_3PbI_{3-x}(SCN)_x$ synthesized with 650mg/ml $Pb(SCN)_2$ solution is presented in Fig 4a. Fig 4a shows the diffraction peaks for the synthesized perovskite appeared at (110), (112), (211), (202), (220), (310), (312), (224), (330).and matches with the reported structure in literature corresponding to incorporation of $(SCN)^-$[3], thus confirming the formation of the $Pb(SCN)_2$ precursor based perovskite. As presented in Fig 4a, the diffraction peaks observed in the XRD pattern of $Pb(SCN)_2$ film are not present in the XRD pattern of the perovskite which confirms the complete conversion of $Pb(SCN)_2$ into perovskite.

Raman Spectrum of $CH_3NH_3PbI_{3-x}(SCN)_x$ film synthesized with 650mg/ml $Pb(SCN)_2$ solution is presented in Fig 4c. A peak corresponding to Raman shift at 149 /cm is observed for $Pb(SCN)_2$ film (Fig 4c). However, the peak observed in the Raman Spectra of $Pb(SCN)_2$ film was not present in the Raman Spectra of the synthesized perovskite film which implies $Pb(SCN)_2$ has been completely converted into the perovskite. The Raman spectroscopy of the perovskite matches with the reported Raman spectra of perovskite with incorporation of $(SCN)^-$[3] and hence confirms on the formation of the $CH_3NH_3PbI_{3-x}(SCN)_x$ perovskite material synthesized with 650mg/ml $Pb(SCN)_2$ solution.

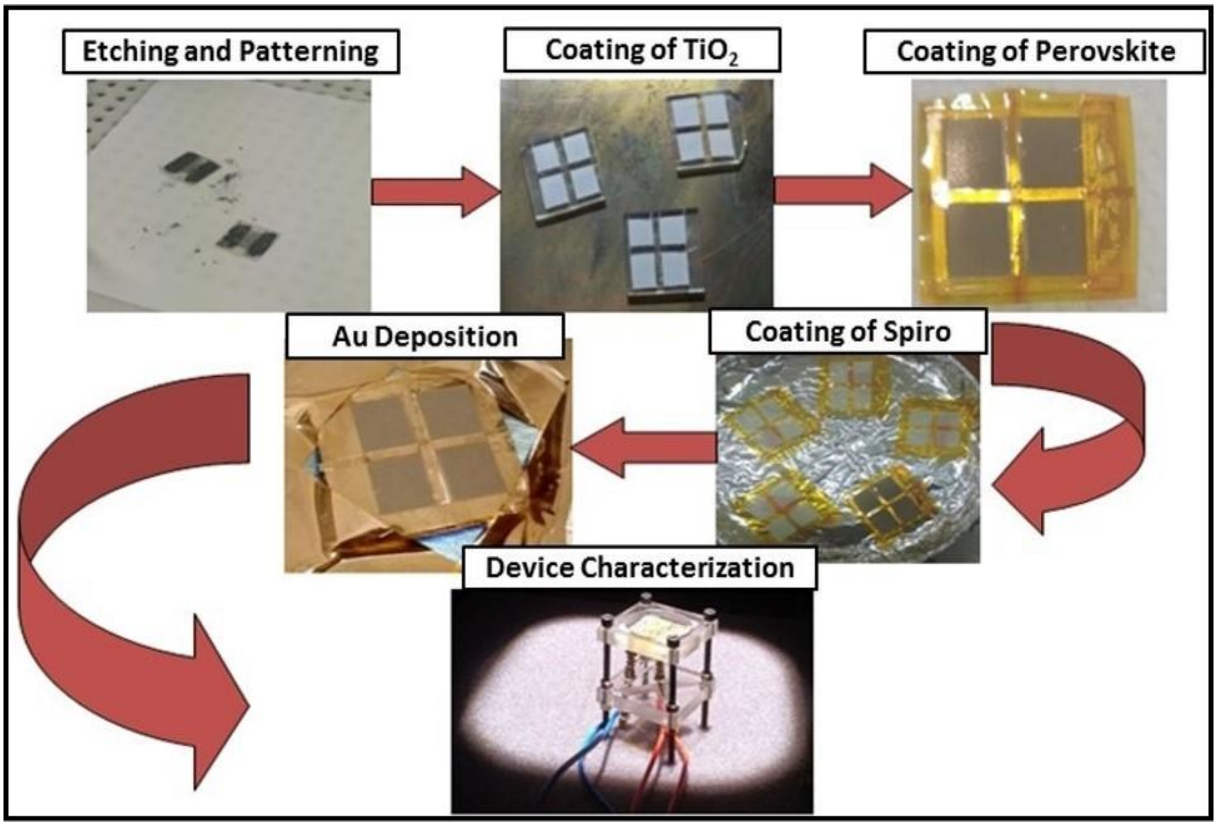

Figure S1. n-i-p PSC fabrication steps

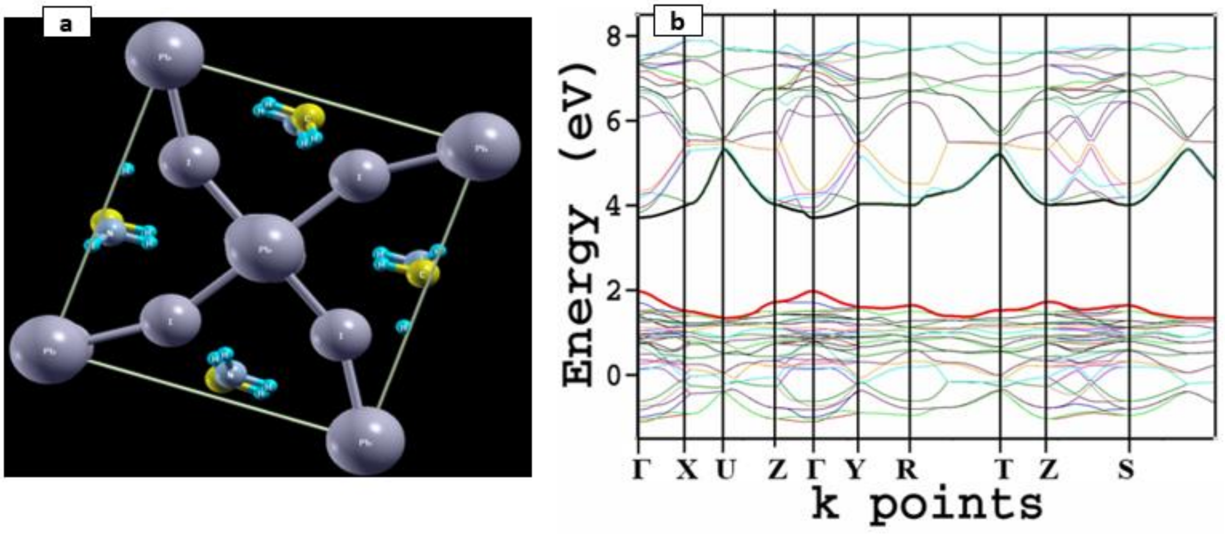

Figure S2. DFT calculated (a) variable cell optimized structure. The perovskite structures were visualized using free and open-source software Quantum Espresso (https://www.quantum-espresso.org/, Version: 6.4.1), and 'XCrySDen' (http://www.xcrysden.org/, Version: 1.6).

(b) band structure at x=0. Graphs were plotted using free and open-source software Quantum Espresso (https://www.quantum-espresso.org/, Version: 6.4.1), and 'Grace' (https://plasma-gate.weizmann.ac.il/Grace/, Version: 5.1.22)

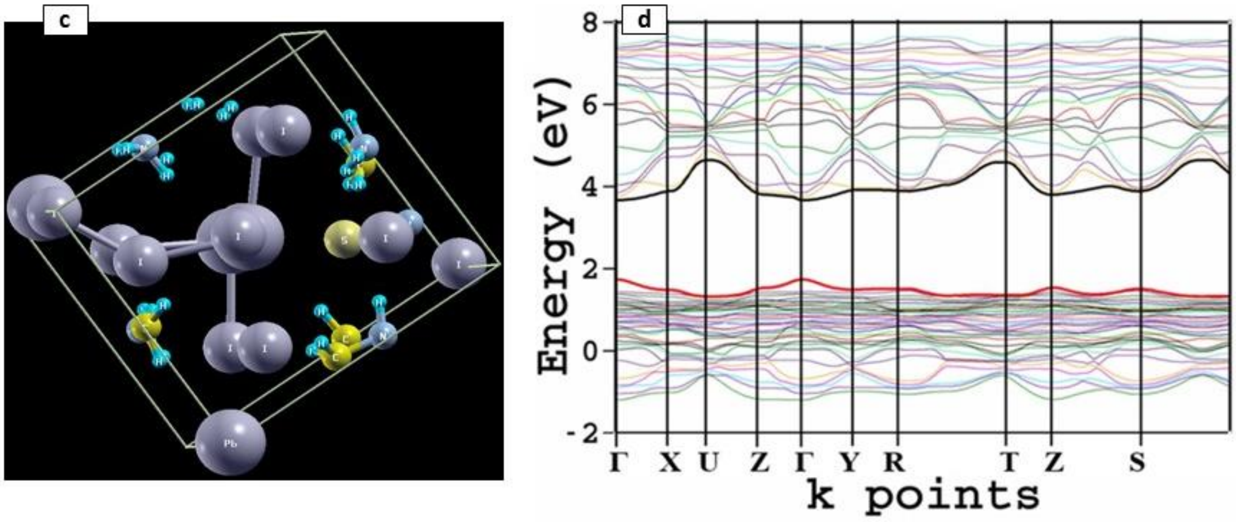

Figure S3. DFT calculated (c) variable cell optimized structure. The perovskite structures were visualized using free and open-source software Quantum Espresso (https://www.quantum-espresso.org/, Version: 6.4.1), and 'XCrySDen' (http://www.xcrysden.org/, Version: 1.6).

(d) band structure at x=0.25. Graphs were plotted using free and open-source software Quantum Espresso (https://www.quantum-espresso.org/, Version: 6.4.1), and 'Grace' (https://plasma-gate.weizmann.ac.il/Grace/, Version: 5.1.22)

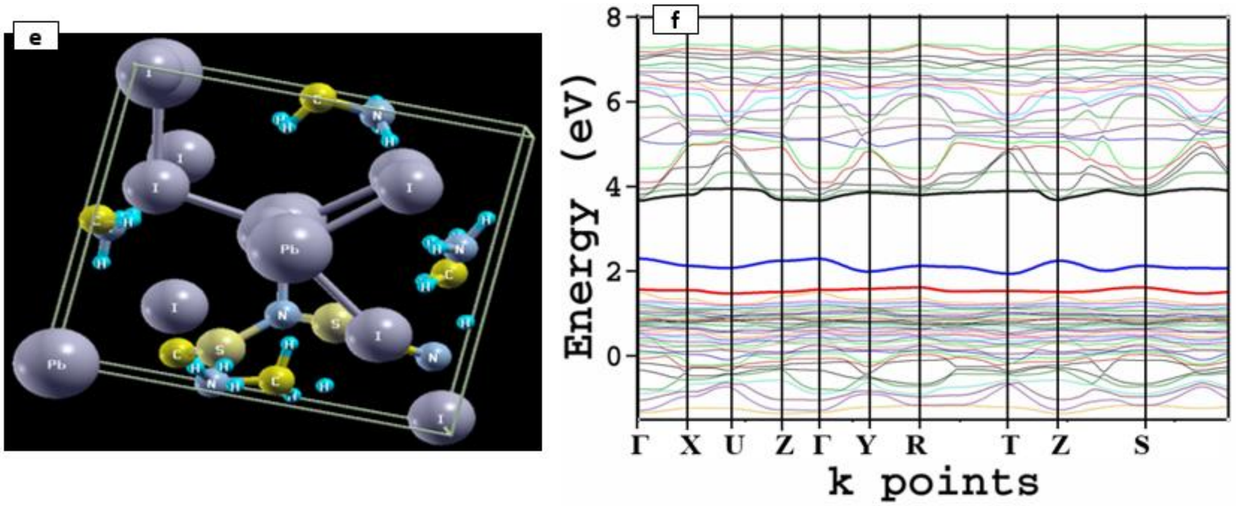

Figure S4. DFT calculated (e) variable cell optimized structure. The perovskite structures were visualized using free and open-source software Quantum Espresso (https://www.quantum-espresso.org/, Version: 6.4.1), and 'XCrySDen' (http://www.xcrysden.org/, Version: 1.6). (f) band structure at x=0.49. Graphs were plotted using free and open-source software Quantum Espresso (https://www.quantum-espresso.org/, Version: 6.4.1), and 'Grace' (https://plasma-gate.weizmann.ac.il/Grace/, Version: 5.1.22)

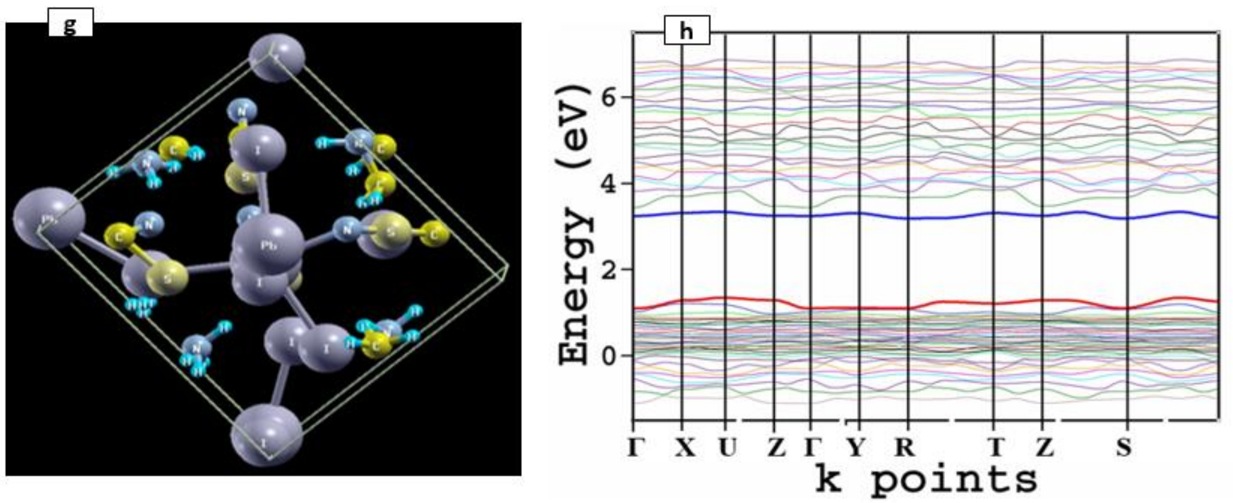

Figure S5. DFT calculated (g) variable cell optimized structure. The perovskite structures were visualized using free and open-source software Quantum Espresso (https://www.quantum-espresso.org/, Version: 6.4.1), and 'XCrySDen' (http://www.xcrysden.org/, Version: 1.6).

(h) band structure at x=1.0. Graphs were plotted using free and open-source software Quantum Espresso (https://www.quantum-espresso.org/, Version: 6.4.1), and 'Grace' (https://plasma-gate.weizmann.ac.il/Grace/, Version: 5.1.22)

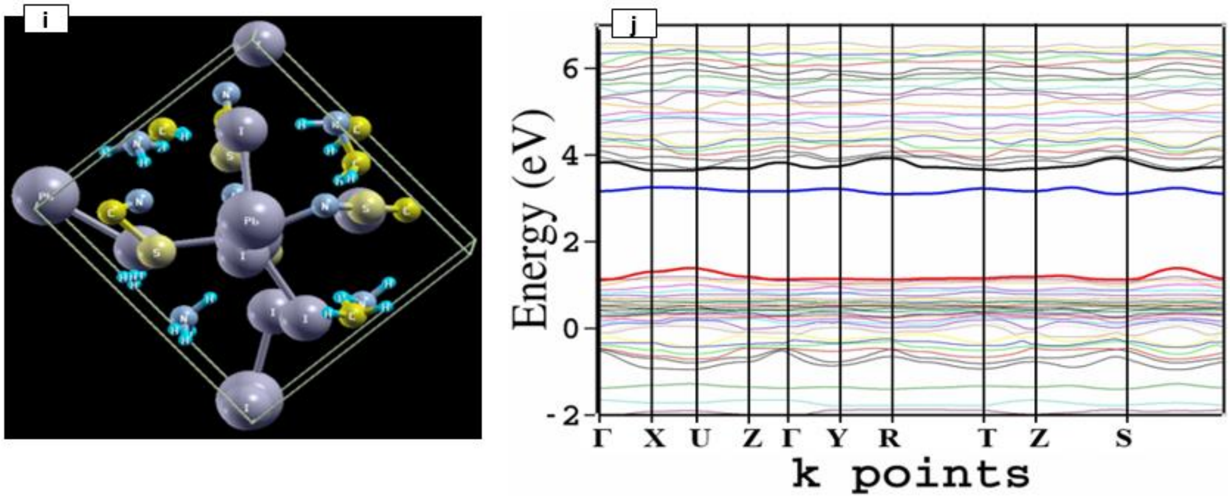

Figure S6. DFT calculated (i) variable cell optimized structure. The perovskite structures were visualized using free and open-source software Quantum Espresso (https://www.quantum-espresso.org/, Version: 6.4.1), and 'XCrySDen' (http://www.xcrysden.org/, Version: 1.6).

(j) band structure at x=1.45. Graphs were plotted using free and open-source software Quantum Espresso (https://www.quantum-espresso.org/, Version: 6.4.1), and 'Grace' (https://plasma-gate.weizmann.ac.il/Grace/, Version: 5.1.22)

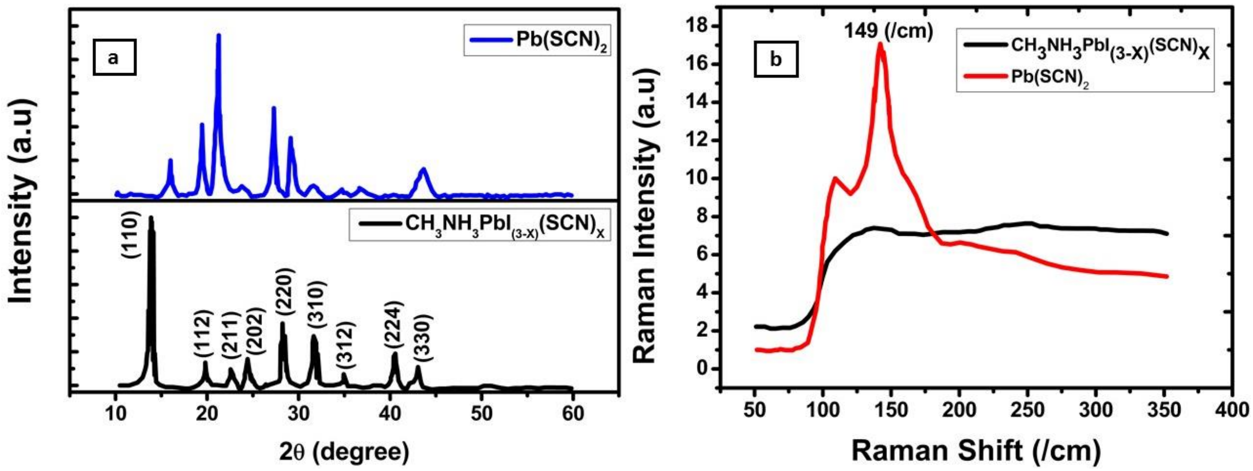

Figure S7. Characterization of $CH_3NH_3PbI_{3-x}(SCN)_x$ at (a) XRD at x=0 (b) XRD at x=1.0 (c) Raman spectra at x=1.0. Graphs were plotted using free and open-source software 'gnuplot' (http://www.gnuplot.info/, Version: 5.2).

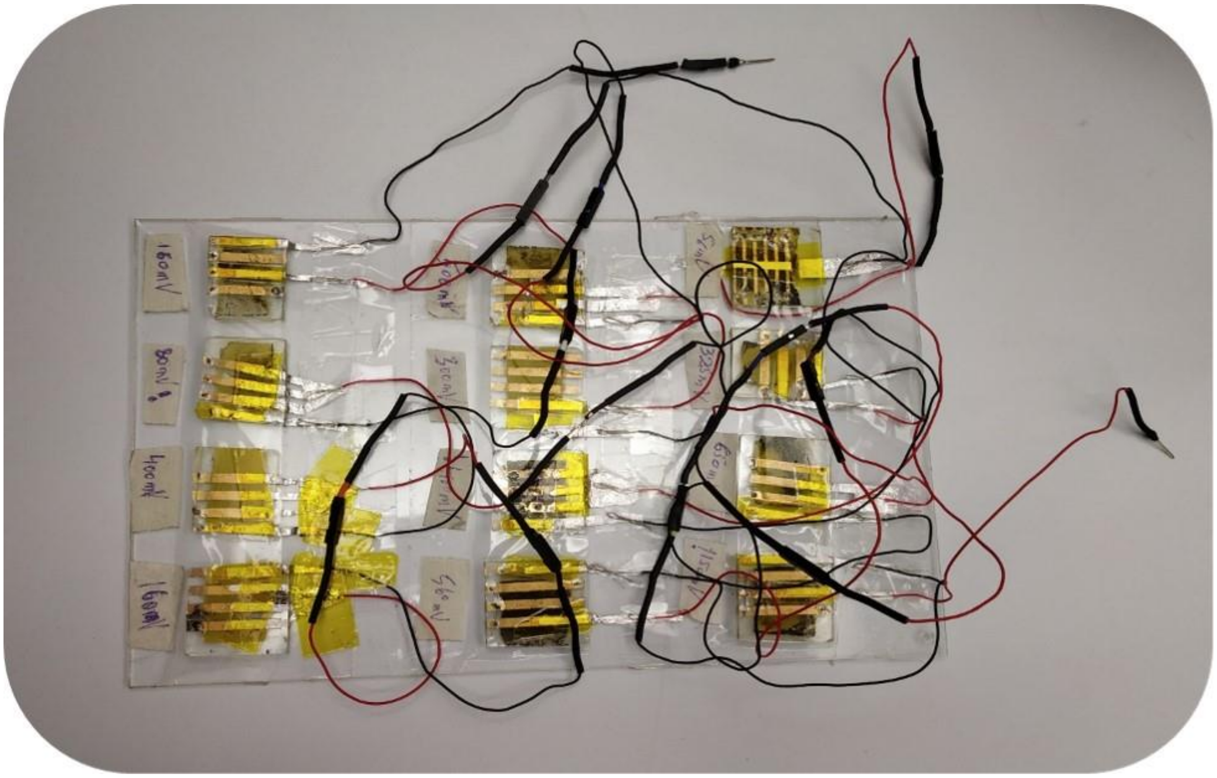

Figure S8. Fabricated solar panel with 36 p-i-n PSCs connected as a combination of series and parallel.
